# Effects of the aqueous extract of a Tibetan herb, *Rhodiola algida* var. *tangutica* on proliferation and HIF-1α, HIF-2α expression in MCF-7 cells under hypoxic condition in vitro

**DOI:** 10.1186/s12935-015-0225-x

**Published:** 2015-08-15

**Authors:** Yu-juan Qi, Sen Cui, Dian-xiang Lu, Ying-zhong Yang, Yushuang Luo, Lan Ma, Yan Ma, Tana Wuren, Rong Chang, Lei Qi, Ba-ji Ben, Jun Han, Ri-Li Ge

**Affiliations:** Qinghai Province people’s Hospital, Xining, 810007 Qinghai Peoples’ Republic of China; Research Center for High Altitude Medicine in Qinghai University, 16 Kunlun Road, Xining, 810001 Qinghai Peoples’ Republic of China; Qinghai University Affiliated Hospital, Xining, 810001 Qinghai Peoples’ Republic of China

**Keywords:** *Rhodiola algida* var. *tangutica*, MCF-7 breast cancer cell, HIF-1α, HIF-2α, Hypoxic

## Abstract

**Ethnopharmacological relevance:**

*Rhodiola algida* var. *tangutica* is a traditional Tibetan herb. Its root and rhizome have been successfully used as an effective clinical remedy for the prevention and treatment of cancer and high-altitude sickness. This study aimed to investigate the effect of *Rhodiola algida* var. *tangutica* on hypoxic MCF-7 breast cancer cells and the underlying mechanisms.

**Materials and methods:**

The antiproliferative effects of *R. algida* on MCF-7 breast cancer cells were compared in vitro under hypoxic and normal conditions by using MTT analysis. The influence of *R. algida* on cancer cell apoptosis was determined by flow cytometry. The expression levels of hypoxia-inducible factor (HIF)-1α and HIF-2α were evaluated by western blot analysis.

**Results:**

*R. algida* inhibited the proliferation of MCF-7 breast cancer cells in a dose- and time-dependent manner. The results of flow cytometry indicated that the antiproliferative effect of *R. algida* was mediated by apoptosis induction. Pretreatment with *R. algida* significantly suppressed the hypoxia-induced proliferation and expression of HIF-1α and HIF-2α in MCF-7 breast cancer cells.

**Conclusions:**

*R. algida* might exert an anti-carcinogenic effect on MCF-7 breast cancer cells by decreasing the protein levels of HIF-1α and HIF-2α, which are overexpressed under hypoxic conditions. This effect might be elicited by inhibiting the hypoxia-induced proliferation of MCF-7 breast cancer cells.

## Background

Breast cancer is the most common malignancy in women in China [1]. Its annual age-standardized incidence estimates was 21.6 per 100,000 women in 2008. The mortality and incidence rates are substantially higher in urban compared to rural areas (35.6 versus 15.5 per 100,000; 6.90 versus 4.60 per 100,000 in 2008) [2]. Breast cancer also seems more aggressive in China than in developed countries, although tumor size and stage at diagnosis decreases year by year [3]. Among women in economically developed Chinese provinces and cities, breast cancer has the highest incidence of all cancers.

Hypoxia is a common feature of solid tumour, most of the cancer cells grow fast and suffer nutrition and oxygen deficiency under hypoxia [4]. Hypoxia response pathways are complicated and offer many options for anticancer treatment. One of the main and relatively hypoxic response pathways involves hypoxia-inducible factor (HIF)-1, which has therefore been studied as a tumor-specific target for anticancer treatment [5, 6]. The HIF DNA binding complex is a heterodimer of alpha and beta subunits, both of which are basic helix-loop-helix transcription factors. The β-subunit of HIF is a constitutive nuclear protein, which is critically involved in a range of transcriptional systems, and was first identified in the context of the xenobiotic response. In cells replete with oxygen, HIF-α subunits are unstable, being rapidly destroyed by the ubiquitin–proteasome pathway. When oxygen tension is lowered, HIF-α subunits are stabilised, translocate to the nucleus, and dimerise with a beta subunit. HIF activation is indeed very common in malignant tumours, and have given insights into the underlying mechanisms. And activates transcription of a range of genes via specific enhancer elements termed hypoxia response elements.

Recently, research has been focused on a homologous member of the HIF family. Both factors, HIF-1 and HIF-2, are regulated in similar ways but have different transactivation domains, implying that they may regulate distinct target genes [7, 8].

The key regulators of hypoxia-induced angiogenesis are the HIF family of protein transcription factors [9]. HIF regulates multiple factors such as VEGF, and nitric oxide synthases to promote tumor growth and angiogenesis. Hypoxic cells undergo a switch from aerobic to anaerobic glucose metabolism. This adaptive response to hypoxia involves the coordinated expression of many genes regulated by HIF, such as those that encode GLUT1, GLUT3, and various glycolytic enzymes [10].

The genus *Rhodiola* L. (Crassulaceae) consists of nearly 200 species in which 20 species are commonly used in traditional medical practice in Eastern Europe and Asia [10, 11]. Of these 20 species, *Rhodiola algida*, *Rhodiola rosea*, *Rhodiola sachalinensis*, *Rhodiola kirilowii*, *Rhodiola crenulata*, *Rhodiola stropupurea*, *Rhodiola qundrifida*, and *Rhodiola sera* are the most effective ones.

In China, *R. crenulata* H. Ohba is the only authorized herb according to Chinese Pharmacopoeia. However, many other species are also popular in folk medicines such as *R. algida*. *Rhodiola algida* var. *tangutica* is a traditional Tibetan herb and grows at an altitude of 3,000–4,500 m in the Qinghai-Tibetan plateau. The medicinal use of the plant is usually concentrated in the roots and rhizomes that contain its main bioactive compounds [12].

Rhodiola plants are prescribed to increase physical endurance, work productivity, longevity, resistance to high latitude sickness, and to treat fatigue, depression, nervous system disorders, etc. [13]. *R. algida* is one of the most effective species of *Rhodiola* L. and has been clinically proven to enhance human immune responses [10, 11]. Despite these findings, the role of *R. algida* as an immunomodulatory agent has been established [14].

Salidroside is regarded as the most important bioactive component and has been used extensively as an indicator for quality evaluation of many Rhodiola species and products [15]. Salidroside and tyrosol were the main component of *Rhodiola algida* var. *tangutica* [16]. Therefore, salidroside was selected in this study as a standard marker to evaluate the quality consistency among different batches of *R. algida*.

The effective components of *Rhodiola* have been studied extensively during recent years; the extract of *Rhodiola algida* var. *tangutica* inhibits division of MCF-7 cells and the cytostatic and antiproliferative effect was related to apoptosis induction mechanism [16]. However, whether it plays an anti-carcinogenic effect under hypoxia condition is yet unclear. The aim of the study is *Rhodiola algida* var. *tangutica* inhibits tumor cell proliferation and transcription factor regulation under hypoxia. In this study, we investigated the pharmacological effect of *Rhodiola* on the proliferation of hypoxic MCF-7 cells. In addition, HIF-1α and HIF-2α expression levels in cultured MCF-7 cells exposed to hypoxia were estimated in vitro.

## Methods

### Reagents

MCF-7 breast cancer cells were purchased from Shanghai Institute for Biological Sciences. The effective components of *R. algida var. tangutica* were isolated and analyzed by high-performance liquid chromatography (HPLC) at the Northwest Institute of Plateau Biology, Chinese Academy of Sciences. The antibodies against HIF-1α, HIF-2α, and glyceraldehyde-3-phosphate dehydrogenase (GAPDH) were purchased from Abcam (UK). Annexin V-FITC was purchased from BD Biosciences (San Jose, CA, USA). Fetal bovine serum (FBS) and antibiotics were purchased from Gibco/RBL (Gaithersburg, MD, USA).All other reagents were made in China.

### Extract preparation

The rhizomes of ground plants of *Rhodiola algida* var. *tangutica* were grown for 3 years in the experimental field of Yushu (altitude 3,800 m) in Qinghai.

The plant material was dried at 80°C. Next, 10 g of the ground dry raw material was mixed with 60 mL of 96% ethanol. Extraction was carried out in flasks placed on a rotary shaker at 25°C in dark for 12 h. The extract was filtered, and the filtrate was evaporated at 35°C, at reduced pressure (20 mbar). The dry residue (0.23 g) was dissolved in 5 mL dimethyl sulfoxide (DMSO) to a final concentration of 2–4.6%. This solution was diluted with distilled water to obtain the following dilutions: 45, 90, 180, 225, 360, and 450 μg/mL; the maximal concentration of DMSO in the obtained dilutions did not exceed 0.5%. A solution of 0.5% DMSO was used as a control in all our experiments.

### HPLC analysis

HPLC analysis was performed using a Phenomenex Kromasil C18 column (5 m, 250 mm × 460 mm), equipped with a UVD detector. Separation was performed using an EC 250/4 Nucleosil^®^ 120-7C18 column (Machery-Nogel). The mobile phase consisted of methanol (A) and 0.01 M H_3_PO_4_ (B), used in the following gradient elution: from 5% A for 0 min to 5% A for 10 min to 100% A for 60 min. The flow rate was 1 mL/min, and the detection wavelength was 220 nm. The results of our investigations determined that the main component of *Rhodiola algida* Var. *tangutica* contained salidroside and tyrosol [16]. The standard substances, i.e., salidroside [15], tyrosol, and other fractions were analyzed in the same manner. The standard substances purchased from China’s food and drug verification research institute (110818–201206). Peaks were assigned by spiking the samples with standard substances and comparing the retention times and UV spectra.

### Cell culture

Cells were cultured in RPMI 1640 containing 10% (v/v) fetal bovine serum (FBS, Gibco) with 100 U/mL penicillin and 100 U/mL streptomycin at 37°C. The fourth to sixth generation cells were used for the subsequent experiments. Cells were exposed to normoxia (74% N_2_, 5% CO_2_, and 21% O_2_) and hypoxia (93% N_2_, 5% CO_2_, and 2% O_2_).

### Morphological changes

The cells were visually and microscopically inspected each time they were handled. Signs of deterioration of cells included granularity around the nucleus, detachment of the cells from the substrate, and cytoplasmic vacuolation.

### Viability studies

MCF-7 cells were incubated in a metabolic water bath for 72 h under normoxic condition. Control tubes contained only cells and incubation medium with DMSO and experimental tubes contained cells in medium along with different concentrations of *R. algida* extract (45, 90, 180, 225, 360, and 450 μg/mL). One tube from each of the 6 experimental sets was withdrawn at 0, 6, 12, 24, 48, and 72 h after incubation and immediately assessed for viability by the trypan blue exclusion method.

### Proliferation assays

The proliferation of MCF-7 breast cancer cells was measured using 3-(4, 5-dimethylthiazal-2-yl)-2, 5-diphenyltetra-zoliumbromide (MTT) assay by colorimetrically measuring the formation of formazan dye. Briefly, MCF-7 breast cancer cells were seeded at a density of 10,000 cells/cm^2^ in 96-well plates. The cells were incubated for 24 h in serum-free RPMI1640 and then exposed to *R. algida* extract (0, 45, 90, 180, 225, 360, and 450 μg/mL) for 48 h under normoxia or hypoxia condition, namely, N48 h or H48 h group, respectively. Plain RPMI1640 was used as the negative control. At the end of treatment, MTT (0.25 mg/mL, Sigma, USA) was added to the plates, and incubation was continued for another 4 h at 37°C. The supernatant was then carefully removed, and 150 µL of DMSO was added to dissolve the formazan crystals. The absorbance of the solubilized product was read at 490 nm (A490) by using an ELISA reader (Bio-Rad, USA). All determinations were replicated at least three times.

The effects of hypoxia on MCF-7 cells were investigated by measuring cell proliferation by direct cell counting. Cells were seeded at a density of 10,000 cells/cm^2^ in 12-well plates and incubated in serum-free RPMI1640 for 24 h. They were then stimulated with *R. algida* extract at 0, 45, 90, 180, 225, 360, and 450 μg/mL. After 48 h, they were washed with phosphate buffered solution, harvested by mild trypsinization, and counted using a hematocytometer. The experiments were conducted under both hypoxic and normoxia conditions.

### Flow cytometry

The mode of cell death via apoptosis and or necrosis produced by the extract treatment in MCF-7 cells was determined by flow cytometry after staining the cells with Annexin V-FITC and PI. Cells were stimulated with *R. algida* extract at 0, 45, 90, 180, 225, 360, and 450 μg/mL for 48 h under normoxia or hypoxia. Treated cells were washed twice with cold PBS and then resuspended in binding buffer at a concentration of 1 × 10^5^ cells/mL. Next, 5 µL of Annexin V-FITC and 10 µL of propidium iodide were added to suspended cells. After incubation for 10 min at room temperature in the dark, the percentage of apoptotic cells was calculated using a flow cytometer (Becton–Dickinson, USA) equipped with argon laser having an emission spectrum of 488 nm. The percentages of apoptotic and necrotic cells were evaluated.

### Determination of HIF-1α and HIF-2α expression by western blot analysis

MCF-7 cells were exposed to normoxia or hypoxia in the absence or presence of *R. algida* extract in the indicated concentrations. Cells were lysed in whole lysis buffer (0.1% Nonidet P-40, 5 mM ethylenediaminetetraacetic acid (EDTA), 50 mM Tris (pH 8.0), 250 mM NaCl, and 50 mM NaF), and protein concentrations were determined using the Bio-Rad Protein assay (Bradford). Subsequently, 30 μg protein was resolved on 10% sodium dodecyl sulfate–polyacrylamide gel electrophoresis (SDS-PAGE) and transferred to nitrocellulose membranes. The membranes were blocked with 5% skimmed milk–PBS/0.1% Tween20 for 2 h before an overnight incubation at room temperature with primary antibodies in 5% skimmed milk in PBS/0.1% Tween20. The corresponding secondary antibody was washed with Tris-buffered saline with Tween20 (TBST) for 30 min and added to the membranes, followed by incubation at room temperature for 2 h. The membranes were then washed three times for 10 min each with TBST. Detection was performed using the enhanced chemiluminescence system (Amersham, Arlington Heights, IL, USA). The relative expression levels of HIF-1α and HIF-2α were normalized against that of GAPDH.

### Statistical analysis

The statistical significance of differences between groups was evaluated by one-way analysis of variance (ANOVA), followed by Dunnett’s test for multiple comparisons. A *p* value of < 0.05 was considered as statistically significant.

## Results

### Effect of hypoxia on MCF-7 cell proliferation

MCF-7 breast cancer cells were cultured under hypoxic or normoxic condition for 12, 24, 48, and 72 h, as described previously. Subsequently, cell proliferation was measured by the MTT assay. The absorbance at 490 nm was detected. Hypoxia-induced MCF-7 breast cancer cells showed proliferation especially at 48 h (Table 1). Therefore, 48 h of incubation was used for the subsequent experiments.

**Table 1:** Effect of hypoxic on proliferation of MCF-7 breast cancer cells (absorbance at 490 nm, A490)

	12 h	24 h	48 h	72 h
Normoxia	0.272 ± 0.023	0.289 ± 0.019	0.315 ± 0.031	0.331 ± 0.003
Hypoxia	0.298 ± 0.031	0.319 ± 0.044^a^	0.351 ± 0.034^a^	0.362 ± 0.044^a^

The data represented mean ± S.D. (n = 15).

^a^ *P* < 0.05 vs. normoxia.

### In vitro viability studies on cytotoxicity of *R. algida* var. *tangutica*

**Fig. 1:**
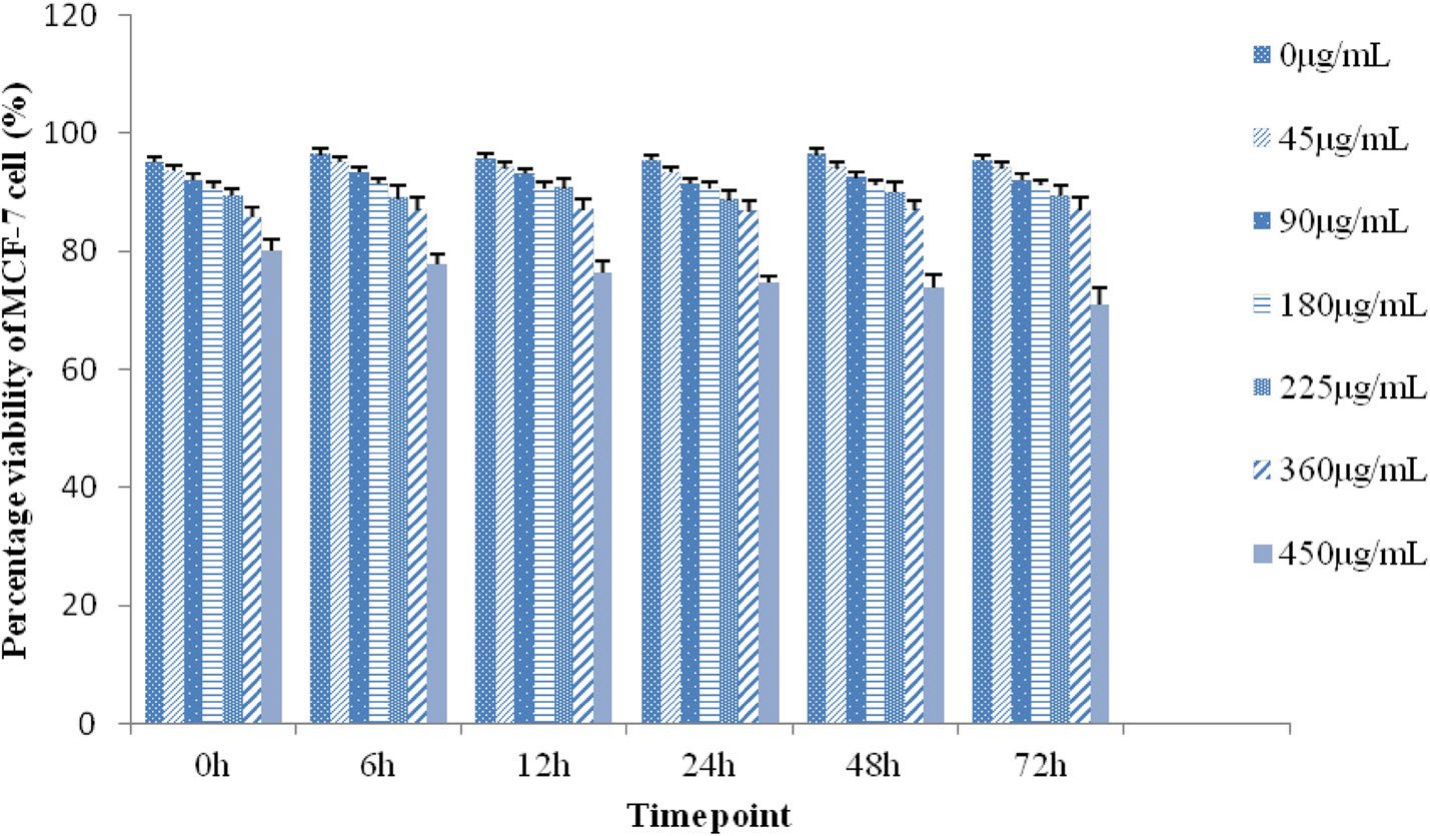
Effect of *Rhodiola algida* var. *tangutica* on the viability of MCF-7 cell. MCF-7 cell were cultured in microwells and incubated with increasing concentrations of *Rhodiola algida* var. *tangutica* 0, 6, 12, 24, 48 and 72 h after incubation with different concentrations of *Rhodiola algida* var. *tangutica*, percentage viability of MCF-7 cell was determined by trypan blue exclusion. The highest concentration (450 μg/mL) of *Rhodiola algida* var. *tangutica* caused a significant decrease in cell viability compared with control.

The cytotoxicity of *R. algida* extract on MCF-7 cells was evaluated by the trypan blue dye exclusion method, and the appropriate concentrations of the plant extract to be used was determined. The MCF-7 (2 × 10^5^ cells/well) breast cancer cells were incubated for 24 h in 96-well plates with various concentrations of *R. algida* extract (0, 45, 90, 180, 225, 360, and 450 μg/mL). Dose-dependent cytotoxic effect of the plant extract against MCF-7 breast cancer cells is shown in Fig. 1. The concentration of 450 μg/mL significantly decreased the number of viable cells and produced microscopic changes in morphology compared with those of the controls. Hence, the doses of 360 μg/mL or less were selected for subsequent experiments since these doses had weak cytotoxic effects on MCF-7 cells. However, with increasing incubation times, even low doses of *R. algida* extract significantly decreased cell viability.

### Morphology

**Fig. 2:**
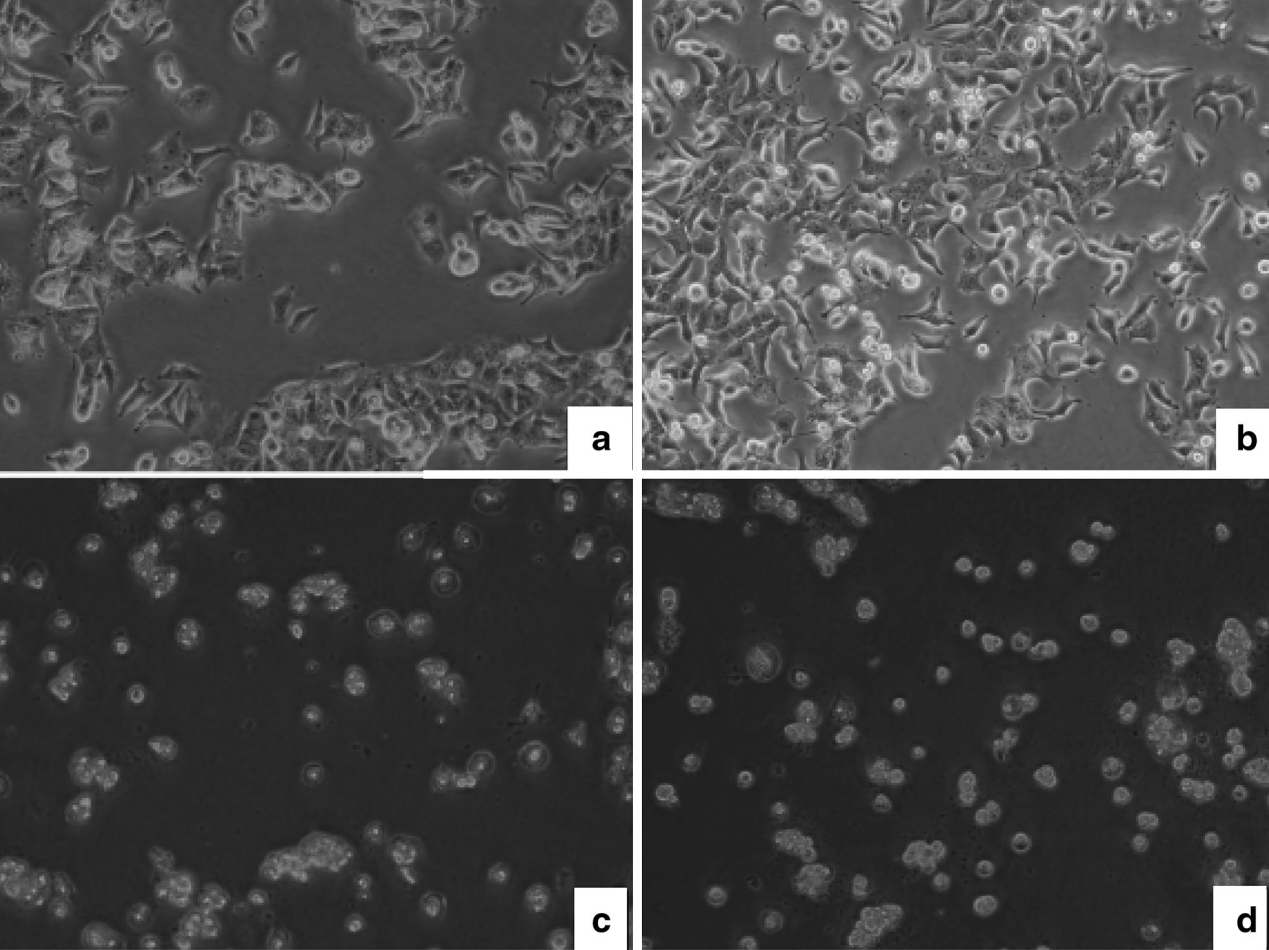
*Rhodiola algida* var. *tangutica* inhibits hypoxia-induced proliferation in MCF-7 cells. The cells were plated at a seeding density of 5 × 10^4^ viable cells/cm^2^ and grown as a monolayer in a 37°C incubator with a humidified atmosphere of 5% CO_2_ in a 2.0% O_2_ or normoxic. The images were obtained using 20× objectives 48 h after plating. **a** Morphology from normal MCF-7 breast cancer cells in adherent culture exposed to normoxic 48 h (N48h). **b** Morphology from MCF-7 breast cancer cells in adherent culture exposed to hypoxia 48 h (H48h), showing proliferation after exposure to hypoxia. **c** Morphology from MCF-7 breast cancer cells in adherent culture receiving *Rhodiola algida* var. *tangutica* (360 μg/mL) for 48 h under normoxic condition (N48h + *Rhodiola algida* var. *tangutica* group), showing shrinkage and chromatin compaction in normal MCF-7 breast cancer cells. **d** Morphology from MCF-7 breast cancer cells in adherent culture receiving *Rhodiola algida* var. *tangutica* (360 μg/mL) after exposure to hypoxia for 48 h (H48h + *Rhodiola algida* var. *tangutica* group).

Morphological changes in MCF-7 breast cancer cells treated with *R. algida* extract under 2.0% O_2_ or normoxic condition were observed microscopically (Fig. 2). The cells showed shrinkage, chromatin compaction, and cytoplasmic vacuolation. Treatment with *R. algida* extract increased the apoptotic cell numbers (Fig. 2). Cell morphology was markedly changed after 48 h incubation with 360 μg/mL *R. algida* extract.

### Identification of apoptosis by flow cytometry analysis

**Fig. 3:**
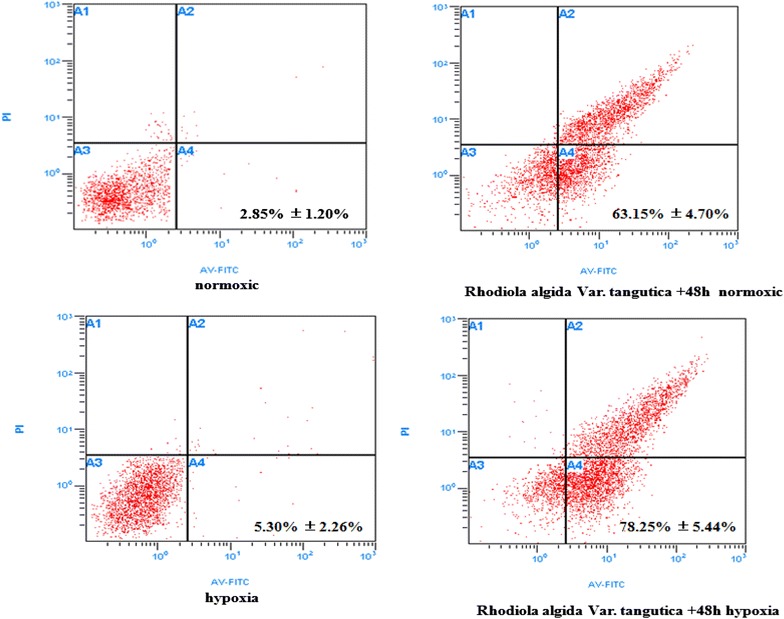
Induction of apoptosis and/or necrosis assessed by flow cytometry. Externalization of phosphotidylserine (PS) to the outer leaflet from the inner leaflet of the plasma membrane is a hallmark of early apoptosis. The FITC labeled Annexin V binds to PS in presence of calcium ions, resulting in green fluorescence of apoptotic cells. In later stages of apoptosis or necrosis, PI enters the cells and bind to cellular DNA, resulting in red and intense green fluorescence with Annexin V. As shown in the Fig. 3, cells treated with the lower concentration showed the present of Annexin V (+) (lower right quadrant) indicating the induction of early apoptosis. However, at higher concentration, there was a prominent rise of Annexin V(+)/PI (+) indicative of late apoptosis/necrosis.

The percentage of MCF-7 cells increased under the hypoxic condition, but this increase in cell proliferation was markedly decreased by treatment with *R. algida* extract (Fig. 3). The data represented mean ± S.D. (n = 15). P < 0.05 vs. normoxia. This suggested that *R. algida* var. *tangutica* induced apoptosis under hypoxia in MCF-7 breast cancer cells.

### Effects of *R. algida* extract on MCF-7 breast cancer cell proliferation under normoxic and hypoxic conditions

**Fig. 4:**
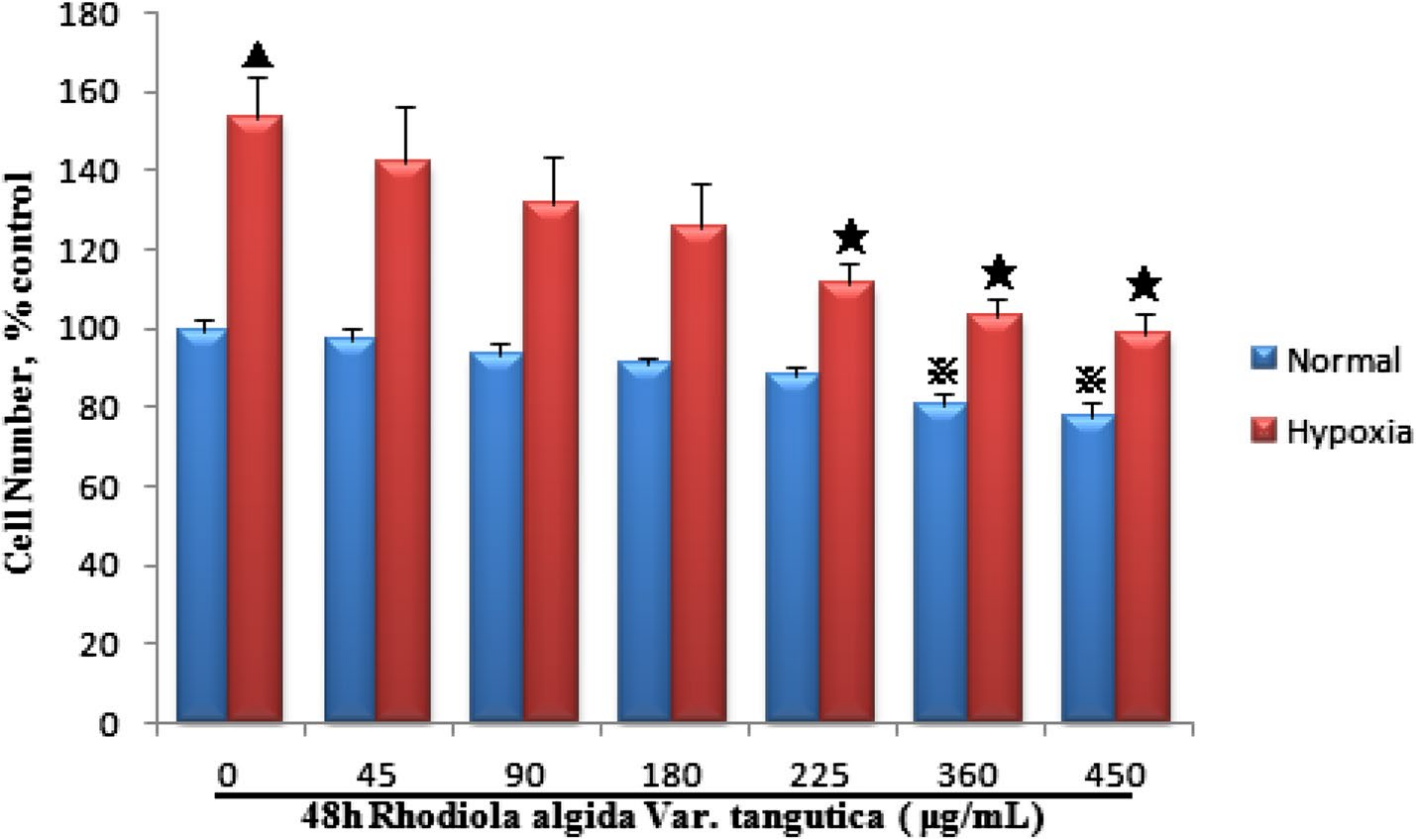
Effect of *Rhodiola algida* var. *tangutica* on proliferation of MCF-7 breast cancer cells exposed to normoxia and hypoxia for 48 h by direct cell counting. Serum-starved MCF-7 breast cancer cells exposed to normoxia and hypoxia were incubated with *Rhodiola algida* var. *tangutica* at different concentrations (0, 45, 90, 180, 225, 360 μg/mL) for 48 h. And then the cell counts were determined with a hematocytometer. Values are expressed as percentage of untreated cells, and each *bar* represents the mean ± S.D. of triplicate determinations. N48 h indicates exposure to normoxia for 48 h; H48 h, exposure to hypoxia for 48 h. ^※^P < 0.05 vs. N48 h in the absence of *Rhodiola algida* var. *tangutica*. ^▲^P < 0.01 vs. N48 h in the absence of *Rhodiola algida* var. *tangutica*; ^★^P < 0.05 vs. H48 h in the absence of *Rhodiola algida* var. *tangutica*.

Evaluation of whether *R. algida* extract affected the proliferation of MCF-7 cells induced by hypoxia was performed using the MTT assay and direct cell counting for nonradioactive quantification of cell proliferation. The results showed that *R. algida* extract inhibited the hypoxia-induced proliferation of MCF-7 cells in a concentration-dependent manner. Significantly reduced cell proliferation of treated cells was also noted under normoxic condition, although at lower concentrations of *R. algida* extract (180 and 225 μg/mL; Table 2). Similar results were obtained by direct cell counting (Fig. 4).

**Table 2:** Effect of *Rhodiola algida* Var. *tangutica* on proliferation of MCF-7 breast cancer cells exposed to normoxia and hypoxia for 48 h (absorbance at 490 nm, A490)

*Rhodiola algida* var. *tangutica*	0 μg/mL	45 μg/mL	90 μg/mL	180 μg/mL	225 μg/mL	360 μg/mL	450 μg/mL
N48 h	0.301 ± 0.070	0.296 ± 0.059	0.284 ± 0.067	0.273 ± 0.058	0.265 ± 0.043	0.247 ± 0.045^a^	0.239 ± 0.062^a^
H48 h	0.358 ± 0.048	0.346 ± 0.054	0.327 ± 0.078	0.314 ± 0.057	0.308 ± 0.071^b^	0.274 ± 0.067^b^	0.266 ± 0.074^b^

The data represented mean ± S.D. (n = 9). N48 h indicates exposure to normoxia for 48 h; H48 h, exposure to hypoxia for 48 h.

^a^P < 0.05 vs. N48 h in the absence of *Rhodiola algida* var. *tangutica*.

^b^P < 0.05 vs. H48 h in the absence of *Rhodiola algida* var. *tangutica*.

### Effect of *R. algida* extract on HIF-1α and HIF-2α protein expression in cultured MCF-7 breast cancer cells

**Fig. 5:**
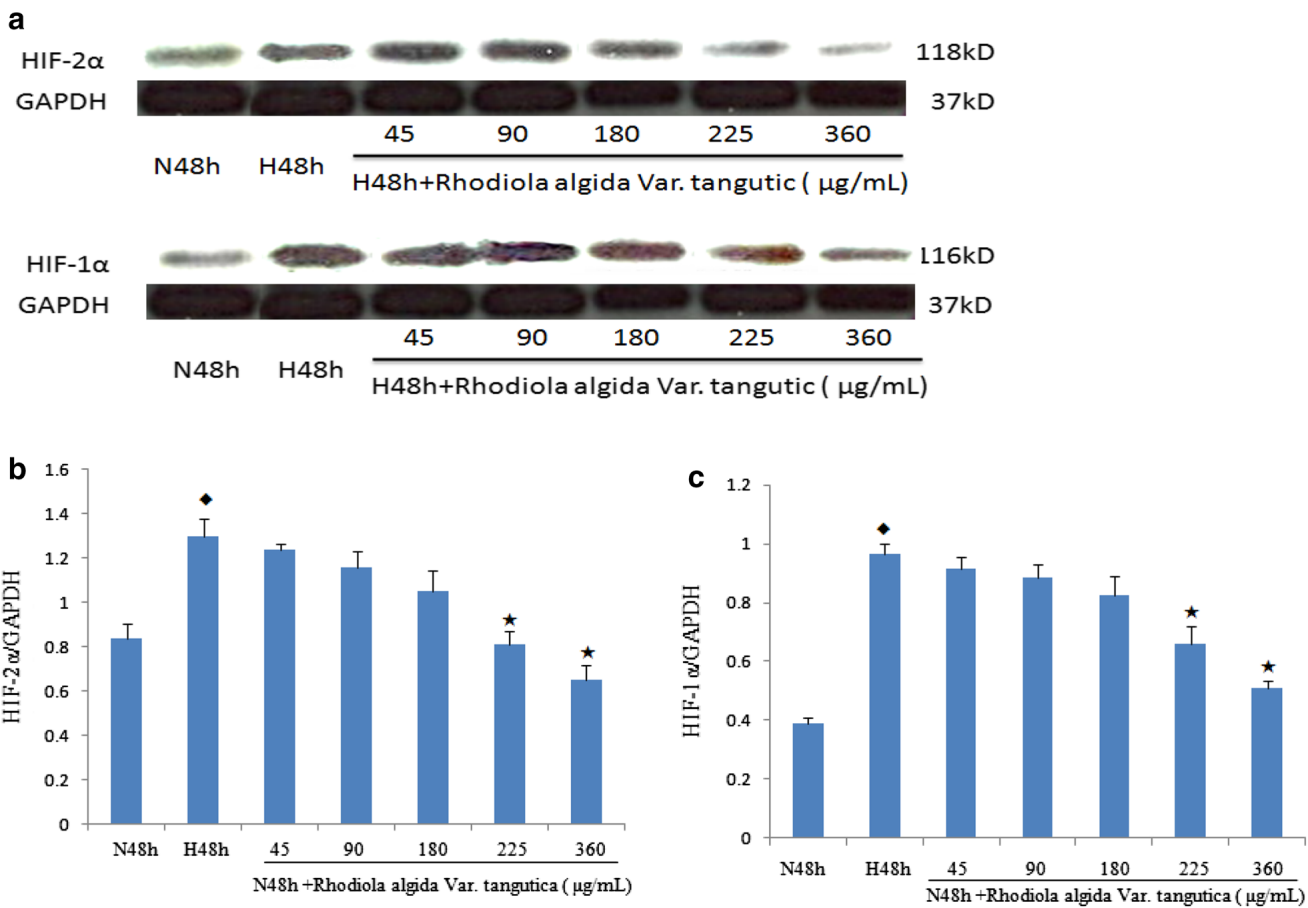
Effects of hypoxia and *Rhodiola algida* var. *tangutica* on HIF-1α, HIF-2α protein expression in MCF-7 cells cultured detected by Western blotting. **a** Western blots of cultured MCF-7 breast cancer cells exposed to hypoxia for 48 h in the absence or presence of various concentrations of *Rhodiola algida* var. *tangutica* (45, 90, 180, 225, 360 μg/mL). Cell lysates were analyzed by Western blotting with antibodies as indicated above. GAPDH protein expression was used as a control. **b**, **c** *Bars* show the relative expression levels of HIF-1α, HIF-2α (optical density of HIF-1α, HIF-2α normalized against GAPDH expression). Data are mean ± SEM of three identical experiments. N48 h indicates exposure to normoxia for 48 h; H48 h, exposure to hypoxia for 48 h. ^◆^P < 0.01 vs. N48 h group, ^★^P < 0.05 vs. H48 h group.

Hypoxia increased the expressionof HIF-1α and HIF-2α in MCF-7 breast cancer cells, whereas treatment with *R. algida* extract (225 and 360 μg/mL) possibly decreased HIF-1α and HIF-2α protein expression in cells exposed to hypoxia for 48 h (Fig. 5, a–c).

## Discussion

Breast cancer has an incidence rate of 16.39 per 100,000 Chinese women and seriously affects the lives and health of this population. Gene targeting involving substances that target hypoxic cells might be an excellent strategy. The transcriptional factor HIF-1 plays an essential role in the adaptive response of cells to reduced oxygen tension [17]. The induction of HIF-1α is a critical step in the induction of hypoxic response and occurs via increased mRNA expression, protein stabilization, and nuclear localization. Additional isoforms of the α-subunit termed HIF-2α and HIF-3α are present. HIF-2α is closely related to HIF-1α, and both subunits can interact with hypoxia-response elements to upregulate their transcriptional activity [18]. In contrast, HIF-3α is involved in the downregulation of the hypoxic response via the activation of an alternatively spliced transcription factor, which may function as an inhibitor of HIF-1α [19]. An efficient gene delivery system needs to be developed to increase the efficiency and reduce toxicity of *Rhodiola* spp. The property of *Rhodiola* spp. to suppress the proliferation of cancer cells under hypoxic condition can be used to induce therapeutic gene expression specifically in the tumor tissue.

Extract of *R.algida* has been known to exert many beneficial effects in the treatment of numerous diseases, probably via different mechanisms reported. For example, salidroside protects endothelial cells from cobalt chloride-induced apoptosis by acting as an antioxidant and regulating the expression of Bcl-2 family members [20]. The protective effect of salidroside has been shown to be closely associated with the activation of the transcription factor HIF-1α along with its target gene *VEGF* [21]. Previous studies have shown that treatment of human peripheral blood mononuclear cells (PBMCs) with *Rhodiola* aqueous extract (RAE) increased the levels of interleukin (IL)-6 and tumor necrosis factor-α (TNF-α) via the phosphorylation of IκB and transcription factor NF-κB; this suggests that RAE has immunostimulatory potential [22]. In some previous study, we found that *R. rosea* aqueous extract, hydroalcoholic extract, and rosavin induced anti-angiogenic activity in the tumor-induced angiogenesis model [23]. Oral administration of *R. rosea* extract to patients with superficial bladder carcinoma led to the improvement in the characteristics of the urothelial tissue integration and T cell immunity [24]. Salidroside induces cell-cycle arrest and apoptosis in human breast cancer cells and may be a promising candidate for breast cancer treatment [25].

The *R. rosea* extract and salidroside inhibit translation initiation. Both the *R. rosea* extract and salidroside treatment of UMUC3 bladder cells caused a significant percentage of cells undergoing autophagy. The *R. rosea* extract and salidroside deserve further study as novel agents for chemoprevention of bladder carcinogenesis [26]. The inhibitory effects of salidroside on tumor metastasis in human fibrosarcoma HT1080 cells in vitro. Salidroside inhibits tumor cells metastasis, which may due to its interfere in the intracellular excess ROS thereby down-regulated the ROS-PKC-ERK1/2 signaling pathway [27]. Angiogenesis was induced in the skin of Balb/c mice by grafting of syngeneic L-1 sarcoma cells. Mice were fed *R. quadrifida* extract or salidroside in daily different doses. After 72 h, *R. quadrifida* extract and salidroside significantly decreased neovascular reaction in all doses applied [23].

In the present study, cultured MCF-7 breast cancer cells exposed to *R. algida* extract at concentrations of 45–360 μg/mL for 48 h showed no signs of toxicity. The effects of hypoxia on the proliferation of MCF-7 breast cancer cells and expression of HIF subunits was also studied. Majewska reported that the rhizome extract of *R. rosea* inhibits cell division, induces apoptosis and necrosis, and elicits cytostatic and anti-proliferative effects in HL-60 cells [28]. Our results are in agreement with previously reported findings and suggest that *R. algida* var. *tangutica* prevents the hypoxia-induced proliferation of MCF-7 cells and down-regulates the expression of HIF-1α and HIF-2α.

## Conclusion

In our research,We found that the proliferation of MCF-7 breast cancer cells increased significantly under hypoxia compared with that of the controls. Our results showed that pretreatment of MCF-7 breast cancer with *R. algida* extract increased cell apoptosis. Moreover, morphological and FACS analyses clearly showed that MCF7 cell proliferation was significantly inhibited by pretreatment with *R. algida* extract under hypoxia. In addition, protein expression of HIF-1α and HIF-2α in MCF-7 cells was inhibited by pretreatment with *R. algida* extract. *R. algida* extract maybe as a potential anti-tumor medicane in the future. Although our experiments were performed in vitro, the results provide a basis for using *R. algida* var. *tangutica* in the treatment of hypoxic breast cancer.

We want to detect the effect of *R. algida* var. *tangutica* on tumor in vivo in our future research job.

## Abbreviations

HIF: hypoxia-inducible factor
VEGF: vascular endothelial growth factor
bFGF: basic fibroblast growth factor
GAPDH: glyceraldehyde-3-phosphate dehydrogenase
DMSO: dimethyl sulphoxide
EDTA: ethylenediaminetetraacetic acid
SDS-PAGE: sodium dodecyl sulfate–polyacrylamide gel electrophoresis
ANOVA: analysis of variance
IL: interleukin
TNF-α: tumor necrosis factor-α
